# QTL Pyramiding and Its Use in Breeding for Increasing the Phytoextraction Efficiency of Soil Cd via High-Cd-Accumulating Rice

**DOI:** 10.3390/plants11162178

**Published:** 2022-08-22

**Authors:** Tadashi Abe, Masashi Ito, Ryuichi Takahashi, Toshimitsu Honma, Masato Kuramata, Satoru Ishikawa

**Affiliations:** 1 Institute for Agro-Environmental Sciences, National Agriculture and Food Research Organization (NARO), Tsukuba 305-8604, Japan; 2 Akita Prefectural Agricultural Experiment Station, Akita 010-1231, Japan; 3 Niigata Agricultural Research Institute, Nagaoka 940-0826, Japan

**Keywords:** rice, cadmium, phytoextraction, three-way cross, Cd-accumulating ability, DNA marker-assisted breeding, food safety

## Abstract

Phytoextraction by high-Cd-accumulating rice lacking a functional *OsHMA3* allele is promising for Cd removal from paddy soils. To increase rice Cd extraction efficiency, we developed a new high-Cd variety, TJN25-11. For this, we pyramided a nonfunctional *OsHMA3* allele from a high-Cd variety, Jarjan, and two QTLs for increased shoot Cd concentrations, which were discovered in a mapping population derived from a high-Cd variety, Nepal 555, and a low-Cd variety, Tachisugata. In two Cd-contaminated paddy fields under drained aerobic soil conditions, TJN25-11 presented significantly higher Cd concentrations in the straw and panicles than the OsHMA3-deficient varieties TJTT8 and Cho-ko-koku. Among the varieties, TJN25-11 had a relatively high shoot biomass, resulting in the highest Cd accumulation in the shoots. The soil Cd decreased by approximately 20% after TJN25-11 growth. The amount of Cd that accumulated in the TJN25-11 aerial parts was much greater than the amount of Cd that decreased in the topsoil, suggesting that Cd was absorbed from deeper soil layers. Thus, we revealed the effects of QTL pyramiding on shoot Cd accumulation and Cd phytoextraction efficiency. Since TJN25-11 has favorable agronomic traits for compatibility with Japanese cultivation systems, this variety could be useful for Cd phytoextraction in Cd-contaminated paddy fields.

## 1. Introduction

Cadmium (Cd) is a ubiquitous element on Earth. Additionally, arable lands contain a small amount of Cd, and crop plants absorb Cd from the soil and accumulate it in their edible parts. Chronic intake of Cd by humans through foods can cause health problems such as renal dysfunction [1]. Therefore, the Codex Alimentarius Commission of the FAO/WHO has set standard values of Cd for various foods to protect our health [2].

Techniques for suppressing Cd uptake by crop plants from the soil have been extensively studied [3,4]. Phytoremediation is an attractive technique for reducing Cd risk in crop plants; in this technique, high-Cd-accumulating (henceforth, high-Cd) plants are used to extract Cd from soils with low-to-moderate levels of Cd contamination [5,6]. The advantages of this method include its cost-effectiveness and environmental friendliness [7]. However, phytoextraction is difficult, as soils contain more Cd than plants can absorb, and Cd-hyperaccumulating plants are needed to increase phytoextraction efficiency [6]. We previously demonstrated that high-Cd rice varieties grown under drained aerobic conditions, which increase soil Cd bioavailability, are the most promising plants for phytoextracting Cd from Cd-contaminated paddy fields [8,9]. Another advantage of the use of high-Cd rice in paddy fields is that it allows farmers to use their own cultivation techniques and machinery [5,9]. Murakami et al. reported that phytoextraction involving the use of the high-Cd rice variety Cho-ko-koku reduced the Cd concentration in the soil by 38% after approximately two years of cultivation and reduced the grain Cd concentration in an edible japonica rice variety by 47% after Cd phytoextraction [10]. If genetic research can lead to further increases in the ability of high-Cd rice varieties to phytoextract Cd from the soil, the time needed for and costs of soil remediation could be reduced.

Owing to genetic differences governing the differential accumulation of Cd in rice plants, the genes involved in rice Cd transport have been identified and functionally analyzed [11]. The high-Cd rice varieties Jarjan, Anjana Dhan, and Cho-ko-koku were identified from among diverse rice accessions [12]. These varieties exhibited high Cd accumulation in their shoots due to a defect in the function of the OsHMA3 protein, a transporter belonging to the P_1B_-type ATPase family, which is localized on the tonoplast, to sequester Cd in the roots [13,14,15]. However, these indica varieties are not amenable for Cd phytoextraction in Japanese paddy soils due to their seed shattering and susceptibility to lodging [16]. Therefore, several high-Cd rice varieties with improved shattering and lodging were developed through DNA marker selection using the loss-of-function allele of *OsHMA3* or mutagenesis, and the ability of these varieties to extract Cd from soils was evaluated [16,17,18,19]. For example, the rice line TJTT8, in which the *OsHMA3* allele of Jarjan was introduced into the genetic background of Tachisugata, which was developed for whole-crop silage, showed higher Cd extraction capacity than Jarjan or Cho-ko-koku on several Cd-contaminated fields [16]. In addition, TJTT8 showed less shattering and lodging resistance and exhibited high local adaptability, which could make it a useful Cd phytoextractor for Cd-contaminated paddy fields.

Ueno et al. selected the indica rice variety Nepal 555 as a high-Cd-accumulating accession and detected two major quantitative trait loci (QTLs) responsible for the high-Cd phenotype [20]. Both QTLs were detected in chromosome regions where *OsHMA3* was not present, indicating that the high Cd accumulation in Nepal 555 is not due to the loss of function of OsHMA3. This may allow the breeding of rice plants with superior Cd accumulation by pyramiding QTLs or alleles involved in high Cd accumulation, which is expected to increase the efficiency of Cd phytoextraction from the soil.

In this study, a rice variety pyramiding the *OsHMA3* allele from Jarjan and QTLs from Nepal 555, both of which accumulate high amounts of Cd in their shoots, was developed through three-way crosses with Tachisugata. The developed variety, named TJN25-11, presented significantly higher Cd in shoots than TJTT8 and Cho-ko-koku, confirming the QTL pyramiding effect of phytoextracting soil Cd by rice plants grown in Cd-contaminated paddy fields.

## 2. Results

### 2.1. Agronomic Traits and Cd Accumulation Ability of Parental Varieties

We characterized several agronomic traits of three parental varieties grown in two Cd-polluted fields in different regions of Japan (Table 1). The soils of both fields are classified as mottled gley lowland soils (Supplementary Table S1). The soil texture was a sandy loam in both fields, but field A contained more sand than field B. The soil Cd concentration extracted by 0.1 M hydrochloric acid (HCl) was higher in field B than in field A.

The days to heading (DH) of Jarjan, Nepal 555, and Tachisugata grown in field A were 110 days, 104 days, and 107 days, respectively. The DH of the three varieties grown in field B were slightly longer than those grown in field A, but varietal differences in DH showed similar trends. In field A, the culm length was longest for Nepal 555, followed by those for Jarjan and Tachisugata. When grown in field B, Jarjan and Nepal 555 had similar culm lengths, with Tachisugata having the shortest culm length. Nepal 555 was susceptible to lodging at maturity. Jarjan and Nepal 555 were susceptible to shattering at maturity, while Tachisugata was resistant.

The Cd concentrations of the straw and grain of the three parental varieties grown in the two Cd-polluted fields were analyzed, and the total amounts of Cd that accumulated in the aerial parts were calculated by multiplying the Cd concentrations by the plant dry weight (Table 1). In field A, the average Cd concentration in the straw was the highest for Jarjan, followed by Nepal 555, and it was lowest for Tachisugata, although there were no significant differences between the varieties due to the large standard deviation. On the other hand, the average grain Cd concentration of Jarjan was four times greater than that of Tachisugata and twofold greater than that of Nepal 555, and a significant difference was detected between Jarjan and Tachisugata. Similarly, in field B, the average straw Cd concentration of Nepal 555 was between those of Jarjan and Tachisugata, and the Cd concentration in the grain was the same as that for Tachisugata. The Cd concentration ratio of grain/straw differed greatly between Jarjan and Napal 555; in field A, Jarjan exhibited greater shoot-to-grain Cd transfer than Nepal 555. Thus, different Cd accumulation profiles in plant tissues were observed between Nepal 555 and Jarjan, indicating that Cd accumulation in Nepal 555 may be regulated by a genetic process different from that of Jarjan. Consistent with Cd concentrations in rice straw, the Cd accumulation in aerial parts was the highest for Jarjan, followed by Nepal 555, and it was lowest in Tachisugata.

### 2.2. Development of TJN25-11 by a Three-Way Cross

**Figure 1:**
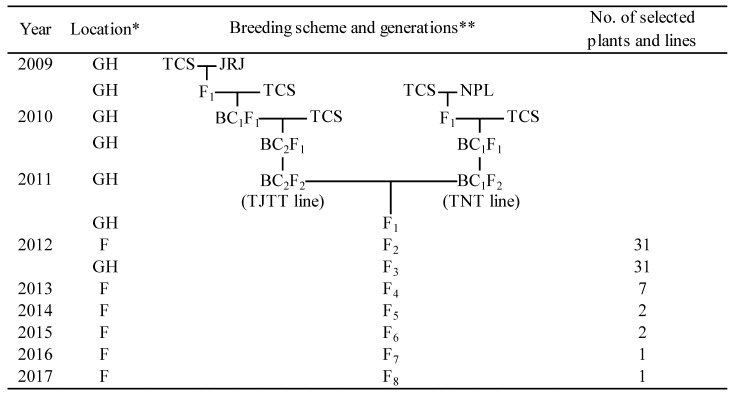
Breeding procedure used in this study. *: GH; greenhouse, F; field. **: TCS; Tachisugata, JRJ; Jarjan, NPL; Nepal555, TJTT line; TCS/JRJ//TCS///TCS (BC_2_F_2_), TNT line; TCS/NPL//TCS (BC_1_F_2_).

**Figure 2:**
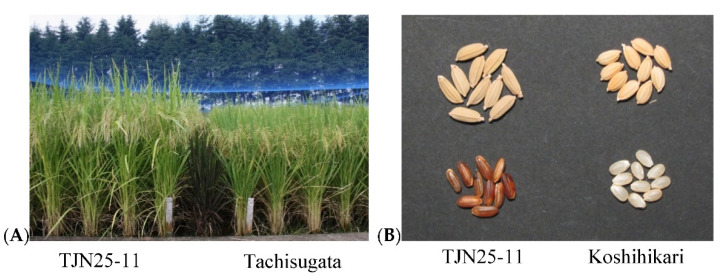
Plant shape (**A**) of TJN25-11 and Tachisugata and grain shapes (**B**) of TJN25-11 and Koshihikari grown in the field.

Figure 1 shows the summarized process used to breed TJN25-11. F_1_ individuals of the TJN lines were obtained by crossing the Tachisugata/Jarjan//Tachisugata///Tachisugata (TJTT; BC_2_F_2_) lines and the Tachisugata/Nepal 555//Tachisugata (TNT; BC_1_F_2_) lines. The F_1_ plants were self-pollinated to obtain an F_2_ population. By the use of marker-assisted selection (MAS) involving simple sequence repeat (SSR) markers (Supplementary Table S2), 31 individuals of the F_2_ population were found to have inherited the *OsHMA3* allele for high Cd accumulation and an *Rc* allele for brown pericarp from Jarjan, two QTLs (henceforth referred to as QTL6 and QTL11) for high Cd accumulation from Nepal 555, and the *qSH1* allele for nonshattering from Tachisugata. The QTL6 and QTL11 used in this study were taken from the results of Ueno et al. (2009) [20]. Because SSR markers to detect QTL6 and QTL11 have been unpublished, primer sequences of the markers are not presented here. After the F_2_ generation, the individuals were repeatedly self-pollinated. In the F_4_ to F_6_ generations, line selection was performed by evaluating the Cd accumulation ability and agronomic traits such as plant shape and lodging resistance of each line grown in the two fields. Figure S1 shows the ratio of the straw Cd concentration of F_4_ progeny of TJN lines to that of Jarjan when grown in field B. Although all TJN lines have the Jarjan-type *OsHMA3* allele and the Nepal 555-type QTLs, not all TJN lines had straw Cd concentrations that were significantly higher than those of Jarjan. The effect of QTL pyramiding on increased Cd concentration was observed in only five lines, namely, TJN11-1, TJN12-2, TJN13-1, TJN14-1 and TJN25-11. Among them, TJN25-11 was selected as a candidate line because it presented high Cd accumulation and good plant shape (Figure 2).

### 2.3. QTL Analysis

Because the effects of QTL6 and QTL11 on increasing Cd accumulation were uncertain in the genetic background of Tachisugata, we performed a QTL analysis of straw Cd concentrations in an F_2_ population derived from a cross between Nepal 555 and Tachisugata. When the F_2_ population of 93 individuals was cultivated in field B, two major QTLs were detected (Table 2). One QTL, named *qHCd2*, was located on the long arm of chromosome 2 (RM1211-RM5303; nearest marker: RM1385), in which the Tachisugata allele was associated with the high Cd concentration in the straw. Another QTL, *qHCd6*, was located on the short arm of chromosome 6 (RM3414-RM2615; nearest marker: RM8258), in which the Nepal 555 allele was associated with the high Cd concentration in the straw. Because QTL6 was detected on the long arm of chromosome 6 [20], *qHCd6* appeared to differ from QTL6. The logarithm of odds (LOD) values were 4.57 for *qHCd2* and 5.76 for *qHCd6*.

TJN25-11 was genotyped using two SSR markers, namely, RM1385 and RM8258, for the detection of *qHCd2* and *qHCd6*, respectively. The RM1385 and RM8258 loci of TJN25-11 were Tachisugata-type homozygotes and Nepal 555-type homozygotes, respectively (Supplementary Figure S3). These results suggest that TJN25-11 has newly identified high-Cd QTLs.

### 2.4. Characteristics of TJN25-11

Table 3, Table 4 and Table 5 show the properties of TJN25-11 grown in two farmers’ fields. The properties of TJN25-11 were compared with those of TJTT8 and Cho-ko-koku. In the field tests, water management was carried out in accordance with the early drainage management method to promote Cd availability in the soil.

#### 2.4.1. Agronomic Traits of TJN25-11

Table 3 shows the agronomic traits of TJN25-11. In field A, the DH of TJN25-11 was 117 days, which was one day later than that of TJTT8 and five days later than that of Cho-ko-koku. In field B, the DH of TJN25-11 was 123 days, which was two days later than that of TJTT8 and eight days later than that of Cho-ko-koku. The culm length of TJN25-11 was 94 cm in field A, which was significantly higher than those of TJTT8 and Cho-ko-koku. In field B, the culm length of TJN25-11 was 102 cm and tended to be higher than those of TJTT8 and Cho-ko-koku, but the difference was not significant. In both fields, the straw weight of TJN25-11 was similar to that of TJTT8 and was significantly higher than that of Cho-ko-koku. The straw weight of TJN25-11 grown in field A was greatest—992 g m^−2^. On the other hand, the panicle weight of TJN25-11 was 332 g m^−2^ for field A and 150 g m^−2^ for field B, similar to that of TJTT8 and significantly lower than that of Cho-ko-koku. TJN25-11 and TJTT8 were resistant to lodging in both fields, whereas Cho-ko-koku was weakly resistant to lodging.

#### 2.4.2. Cd Accumulation Ability of TJN25-11

Table 4 shows the Cd concentration and the amount of Cd accumulated in aerial parts of the plants grown in the two Cd-contaminated fields. In field A, the Cd concentration of the straw of TJN25-11 was 24.5 mg kg^−1^, which was higher than those of TJTT8 and Cho-ko-koku, although the difference was not significant. The Cd concentration of the panicles of TJN25-11 was 37.4 mg kg^−1^ and was three times higher than that of Cho-ko-koku. In field B, the Cd concentrations of the straw and panicles of TJN25-11 were 21.1 mg kg^−1^ and 27.9 mg kg^−1^, respectively, which were significantly higher than those of TJTT8 and Cho-ko-koku. The amounts of Cd that accumulated in the straw and panicles are given in milligrams per square meter and were calculated by multiplying the Cd concentration by the dry weight of each part per square meter. In both fields, the straw and panicles of TJN25-11 accumulated the most Cd compared to those of TJTT8 and Cho-ko-koku. The total amounts of Cd accumulation in the aerial parts (straw + panicles) of TJN25-11 were 36.5 mg m^−2^ in field A and 20.5 mg m^−2^ in field B and were significantly higher than those of TJTT8 and Cho-ko-koku. TJN25-11 accumulated approximately one and a half times to twice as much Cd as TJTT8 or Cho-ko-koku, respectively, in both fields, indicating that the ability of TJN25-11 to accumulate Cd is superior to those of TJTT8 and Cho-ko-koku.

#### 2.4.3. Cd-Phytoextraction Efficiency of TJN25-11

Table 5 shows the reduction in soil Cd concentrations after phytoextraction. The Cd concentrations in topsoil (0–15 cm) were estimated by extraction with 0.1 M HCl, which is the method stipulated by the Agricultural Land-Soil Pollution Prevention Law in Japan, before and after phytoextraction. The soil Cd concentrations in both fields decreased after phytoextraction. TJN25-11 reduced the soil Cd concentrations from 0.33 mg kg^−1^ to 0.26 mg kg^−1^ in field A and from 0.51 mg kg^−1^ to 0.40 mg kg^−1^ in field B. When the actual extraction efficiency of Cd from the soils was calculated from the Cd reduction in soil before and after planting, TJN25-11 presented values of 21.2% in field A and 21.6% in field B, the rates of which were greater than those of TJTT8 and Cho-ko-koku. The theoretical phytoextraction efficiency of soil Cd was also calculated from the total amount of Cd accumulation in the aerial parts, as shown in Table 4. Theoretical calculations based on shoot Cd accumulation yielded phytoextraction efficiency percentages that were seemingly higher than the actual percent reductions in soil Cd concentrations for all varieties, especially in field A.

## 3. Discussion

Previous studies have developed practical high-Cd rice varieties for phytoextraction of Cd from soils by improving traits that are disadvantageous to mechanical harvesting, such as shattering and lodging, of the high-Cd indica varieties Cho-ko-koku and Jarjan [16,17,18,19]. In addition to improving desirable agronomic traits, genetically increasing the ability to accumulate Cd is essential for efficient Cd-phytoextraction practices involving rice. In this study, using MAS, we successfully developed a new high-Cd-accumulating rice variety, TJN25-11, by the introduction of multiple genes associated with high Cd accumulation from Jarjan and Nepal 555 into the genetic background of Tachisugata.

Jarjan, one of the donor parents for the high-Cd-accumulation trait, accumulated more Cd in the straw and panicles as a result of failure by OsHMA3 to sequester Cd into the root cell vacuoles (Table 1) [15,21]. On the other hand, Nepal 555, another donor parent, exhibited a Cd accumulation profile that was different from that of Jarjan; Nepal 555 accumulated more Cd in the straw but less in the panicles with lower shoot-to-grain Cd transfer than Jarjan (Table 1). These phenotypic differences can be explained by the differences in gene loci controlling Cd accumulation between the two varieties. In fact, Ueno et al. (2009) identified two QTLs from Nepal 555, and the *OsHMA3* gene was not present in these QTLs [20]. Moreover, two QTLs had an epistatic effect on shoot Cd accumulation [20], suggesting that both QTLs should be introduced into Tachisugata to obtain a high-Cd phenotype derived from Nepal 555. However, in this study, the epistatic effect was unclear because not all the TJN lines pyramided with target QTLs had straw Cd concentrations that were significantly higher than those of Jarjan (Supplementary Figure S1). QTL detection and effect might be affected by the genetic background of the population, plant age, tissues used for phenotypic evaluation, and experimental conditions [22]. Although Ueno et al. (2009) detected QTLs from Nepal 555, their Cd studies were conducted hydroponically, and young seedlings from the F_2_ population generated from a cross with the Shwe War variety were used [20]. Therefore, it is possible that QTL6 and QTL11 might not be effective in TJN accessions in the genetic background of Tachisugata. In fact, our QTL analysis revealed two new QTLs (*qHCd2* and *qHCd6*) in the F_2_ population derived from Tachisugata and Nepal 555 (Table 2). *qHCd6* appeared to be located in a region different from QTL6 on chromosome 6. Moreover, *qHCd2* was detected on the long arm of chromosome 2 and was located near a QTL that increased the Cd concentration of shoots according to the results of a QTL analysis with the varieties Badari Dhan and Shwe War [23]. Since Tachisugata is a japonica-indica hybrid variety, it is possible that *qHCd2* from Tachisugata is inherited from the high-Cd-accumulating indica variety Milyang 23, which is one of the ancestors of Tachisugata, although the QTLs related to high Cd accumulation in Milyang 23 have not yet been identified.

Since TJN25-11 has two newly found QTLs, the superior Cd accumulation of TJN25-11 may be attributed to these QTLs with the loss of function of the *OsHMA3* allele. In addition to its superior Cd accumulation, TJN25-11 has several favorable traits, such as nonshattering and lodging resistance (Table 3), that are required for practical Cd phytoextraction in Japanese paddy fields. Such traits were obtained from Tachisugata, which was developed for whole-crop silage. The propensity for high shoot biomass of TJN25-11 is also likely to be inherited from Tachisugata. Since shoot Cd accumulation is determined by multiplying the Cd concentration by the dry weight, a high biomass would be advantageous for Cd-phytoextraction. The seeds of TJN25-11 are brown, the trait of which was inherited from the Jarjan-type *Rc* gene; these seeds are easily distinguishable from common Japanese rice seeds, which are white (Figure 2). Because the *Rc* and *OsHMA3* genes are located in close proximity on chromosome 7, it was possible that they were detected simultaneously by the same markers. This seed color difference is necessary for reducing the risk of contamination of edible rice. When TJN25-11 was cultivated in Cd-contaminated fields under drainage conditions, seed fertility was relatively poor, resulting in low grain yield (Table 3). Poor seed fertility may also be an advantage for phytoextraction to prevent seed contamination. We confirmed that the seed fertility of TJN25-11 improved when the plants were grown in submerged conditions, suggesting that TJN25-11 may be sensitive to drought stress. 

To assess the phytoextraction efficiency of Cd from soils, it is necessary to measure not only the amount of Cd accumulated in the shoots but also the amount of Cd removed from the soil. Of the rice varieties tested, the highest soil Cd reductions were observed after growing TJN25-11 (Table 5). This finding supports the highest Cd accumulation in aerial parts being found in TJN25-11, as shown in Table 4. Estimations revealed that TJN25-11 could remove approximately 21% of the initial Cd present in the soil. However, the theoretical removal efficiency calculated from the amount of Cd in the shoots was higher than 21%. In particular, there was a large gap between the actual and theoretical efficiencies obtained in field A. The soil texture of field A is sandy, so the soil is subject to thorough drying after early draining. Under such conditions, rice roots may search for water and penetrate soil layers deeper than the tillage layer (0–15 cm), absorbing Cd along with water from the deeper layers. The rate of decrease in the Cd concentration in the lower soil layers needs to be investigated in the future. In addition, the physiological and molecular mechanisms, including the genes involved in the increased Cd accumulation of TJN25-11, remain unknown and are challenges for the future. We believe that TJN25-11 could be a useful rice variety for phytoextraction in low-to-moderate-Cd-contaminated paddy fields and could contribute to food safety.

## 4. Materials and Methods

### 4.1. Plant Materials

TJN25-11 was developed from a three-way cross derived from Jarjan, Nepal 555, and Tachisugata. The characteristics of each parent variety are as follows: Both Jarjan and Nepal 555 are indica varieties that accumulate high amounts of Cd in their shoots, are easily shattered, are susceptible to lodging, and have brown pericarps. Tachisugata, a japonica-indica hybrid variety, is used for whole-crop silage because of its high biomass, strong culms, and nonshattering properties [24]. The breeding procedure of TJN25-11 is summarized in Table 1. Briefly, to produce TJTT lines, Tachisugata (recurrent parent) was crossed with Jarjan, a donor parent of the high-Cd (the QTL includes the loss-of-function allele of *OsHMA3*) and brown pericarp (*Rc* allele) traits, and the resulting F_1_ plants were crossed twice with Tachisugata. We carried out MAS from the BC_1_F_1_ generation to the BC_2_F_2_ generation to introduce the *OsHMA3* and *Rc* alleles from Jarjan [15,21,25] and the nonshattering-related allele (*qSH1*) from Tachisugata [15,21,25,26]. For MAS, SSR markers RM5389 and RM1316-1 were used for the detection of *qSH1*. Another SSR marker set, RM6728 and RM21320, was used for the detection of both *OsHMA3* and *Rc* alleles (Supplementary Table S2) [27,28]. In the TNT lines, Tachisugata was crossed twice with Nepal 555, a donor parent with two QTLs, namely, QTL6 and QTL11, for high shoot Cd concentrations [20]. We carried out MAS from the BC_1_F_1_ generation to the BC_1_F_2_ generation to introduce QTL6 and QTL11 from Nepal 555 and *qSH1* from Tachisugata [26]. Because the SSR markers used to detect QTL6 and QTL11 have been unpublished, the primer sequences are not presented here. The detection of *qSH1* was performed via the same marker used for the TJTT lines (Supplementary Table S2) [27,28].

The TJN lines were developed by crossing the TJTT lines (BC_2_F_2_) and TNT lines (BC_1_F_2_). In the F_2_ generation, the 31 TJN individuals were selected via MAS involving SSR markers at *OsHMA3* (derived from Jarjan), *Rc* (derived from Jarjan), QTL6, QTL11 (derived from Nepal 555), and *qSH1* (derived from Tachisugata) (Supplementary Table S2). In the F_4_ to F_6_ generations, line selection was performed by evaluating the plant shape and lodging resistance. In the F_6_ to F_8_ generations, the Cd accumulation ability of the candidate lines was tested by growing the plants in paddy fields. Afterward, the individual variability of a promising line was investigated in the F_7_ and F_8_ generations, and genetic fixation was ultimately confirmed.

TJTT8, which is a backcross inbred line derived from Jarjan and Tachisugata, was previously produced for practical Cd phytoextraction use [16]. This variety and Cho-ko-koku, a reference variety for evaluating Cd accumulation ability, were also grown in paddy fields to compare their properties with the properties of TJN25-11.

### 4.2. Field Tests

Field A and field B were farmers’ fields located in the middle and northeastern regions of Japan. Soil samples were collected from each field to a depth of 15 cm from the surface soil before and after planting. Soil physicochemical properties were measured according to the methods of Ishikawa et al. [29]. Rice seedlings were transplanted into each paddy field at the same time in mid-to-end-May under submerged conditions. Irrigation water was withheld around the end of June to promote the availability of Cd in the soil. Afterward, the paddy fields were managed under rain-fed conditions until harvest [16]. Fertilizers consisting of N, P_2_O_5_, and K_2_O were applied to the fields according to the standards of local farmers.

### 4.3. Evaluation of Agronomic Traits

Agronomic traits were evaluated according to the methods of Abe et al. [16]. The number of test plots for evaluation of the parental varieties was replicated twice (two plots), and that of TJN25-11 was replicated three times (three plots). The culm length, panicle length, and number of panicles were measured on 10 or 20 hills for each plot. The yield of straw and grain was measured for six samples collected from each plot to evaluate the parental varieties. The yield of the straw and panicles was measured for 20 hills per plot for the evaluation of TJN25-11. The heading date was considered the day when 50% of the panicles headed. The lodging degree was scored by observations at maturity based on the proportion of lodging individuals out of the total number of individuals: 0 for all plants erect, 1 for <20% of the plants that became lodged, 2 for 20–40%, 3 for 40–60%, 4 for 60–80%, and 5 for 80–100% (few plants erect). Shattering was evaluated by gripping and pulling the maturing panicles by hand at 35–40 days after heading and was categorized as resistant (r), moderate (m), or susceptible (s) based on the percentage of shattering grains, i.e., less than 20%, more than 20% but less than 50%, and more than 50%, respectively.

### 4.4. Cd Analysis

The plant materials were divided into grains or panicles and straw. The samples were dried and ground to a fine powder with a grinder equipped with a titanium-coated blade to avoid the possible contamination of Cd and other metals. Acid digestion and analysis of Cd concentrations in the rice plants were performed by outsourcing analytical services specializing in metal analysis (Industrial Analysis Service, Ltd., Saitama, Japan). A total of 0.5 g of each ground sample was weighed, placed into a polytetrafluoroethylene tube, and digested with 6 mL of 61% HNO_3_ and 2 mL of 30% H_2_O_2_ using a microwave digestion system (MW3000, Anton Paar, Austria). The digested solution was filtered, and the total volume was brought to 50 mL with Milli-Q water. The concentrations of Cd in the plant tissue were determined with an inductively coupled plasma-mass spectrometer (7500 series, Agilent Technologies, Santa Clara, CA, USA).

### 4.5. QTL Analysis of Straw Cd Concentrations

QTL analysis for increased Cd concentrations in the straw was performed according to the methods of Abe et al. (2011) [21]. A mapping F_2_ population consisting of 93 individuals was generated by crossing Tachisugata and Nepal 555 and genotyped by the use of 110 SSR markers (Supplementary Figure S2) [27,28]. For phenotypic evaluations, the population was grown in field B under the same conditions as those described above, and the Cd concentration in the straw was measured. A linkage map was constructed from the genotypic data with MapMaker/EXP version 3.0 [30]. QTL analysis was performed using composite interval mapping by QTL Cartographer version 2.5 (https://brcwebportal.cos.ncsu.edu/qtlcart/ (accessed on 1 April 2013)). Genome-wide threshold values (α = 0.05) were used to detect putative QTLs based on the results of 1000 permutations.

### 4.6. Statistics

Statistical analysis was performed with Ekuseru-Toukei 2006 statistics software (Social Survey Research Information Co., Tokyo, Japan). Tukey tests were conducted to determine significant differences in Cd contents and agronomic traits.

## Figures and Tables

**Table 1:** Agronomic traits and Cd accumulation ability of parental varieties grown under early drainage water management in two farmers’ fields.

	Variety	Days to Heading	Culm Length	Lodging Degree	Shattering Behavior	Cd Concentration		Total Amount of Cd Accumulation in Aerial Parts
						Straw	Grain	
			(cm)	(0–5)	(s-r)	(mg kg^−1^)	(mg kg^−1^)	(mg m^−2^)
Field A	Jarjan	110	109 ^b^	0.0	s	10.0 ^a^ ± 6.0	8.7 ^a^ ± 8.1	8.8 ^a^
	Nepal 555	104	118 ^a^	4.0	s	8.6 ^a^ ± 6.3	2.0 ^ab^ ± 1.5	4.7 ^ab^
	Tachisugata	107	94 ^c^	0.0	r	4.0 ^a^ ± 3.0	1.3 ^b^ ± 1.0	2.1 ^b^
Field B	Jarjan	115	129 ^a^	0.0	s	14.5 ^a^ ± 3.2	4.9 ^a^ ± 1.0	13.7 ^a^
	Nepal 555	107	126 ^a^	3.0	s	10.6 ^b^ ± 2.7	2.2 ^b^ ± 0.5	9.8 ^b^
	Tachisugata	111	101 ^b^	0.0	r	7.2 ^b^ ± 1.5	2.2 ^b^ ± 0.4	6.6 ^c^

The data are the means ± standard deviations. The different letters show statistically significant differences between varieties at the 5% level of significance according to the Tukey–Kramer test (only culm length in field B) or the Tukey test (other data). Lodging degree: 0 for all plants erect, 1 for <20% of the plants that became lodged, 2 for 20–40%, 3 for 40–60%, 4 for 60–80%, and 5 for 80–100% (few plants erect). Shattering was categorized as resistant (r), moderate (m), or susceptible (s) based on the percentage of shattering grains, i.e., less than 20%, more than 20% but less than 50%, and more than 50%, respectively, at 35–40 days after heading. The culm length was measured on 20 hills (although only Nepal 555 in field B was 10 hills). The sample number for Cd concentration and total amount of Cd accumulation were measured in three samples with two hills combined per plot. The experiment was performed on two plots.

**Table 2:** Positions and effects of putative QTLs for high Cd concentration in rice straw.

Chr.	QTL	Marker Interval	Nearest Marker	LOD *	R^2^ **	AE ***
2	*qHCd2*	RM1211-RM5303	RM1385	4.57	18.8	1.05
6	*qHCd6*	RM3414-RM2615	RM8258	5.76	22.8	−1.17

*: After 1000-permutation tests, the threshold values of the LOD for Cd concentrations of straw were calculated as 4.0. **: R^2^ represents the proportion of the phenotypic variance explained by each QTL. ***: A negative value of the additive effect (AE) indicates that the allele from Nepal 555 increased the phenotypic value.

**Table 3:** Agronomic traits of rice varieties grown under early drainage water management in two farmers’ fields.

	Variety	Days to Heading	Culm Length	Straw Weight	Panicle Weight	Lodging Degree
			(cm)	(g m^−2^)	(g m^−2^)	(0–5)
Field A	TJN25-11	117	94 ^a^ ± 3.2	992 ^a^ ± 60	332 ^a^ ± 39	0
	TJTT8	116	81 ^b^ ± 1.3	922 ^a^ ± 9	237 ^a^ ± 53	0
	Cho-ko-koku	112	82 ^b^ ± 3.2	651 ^b^ ± 89	594 ^b^ ± 110	2
Field B	TJN25-11	123	102 ^a^ ± 4.1	777 ^a^ ± 75	150 ^a^ ± 18	0
	TJTT8	121	94 ^a^ ± 1.8	784 ^a^ ± 69	137 ^a^ ± 12	0
	Cho-ko-koku	115	100 ^a^ ± 4.6	613 ^b^ ± 40	328 ^b^ ± 48	3

The data are the means ± standard deviations. The different letters show statistically significant differences between varieties at the 5% level of significance according to the Tukey test. Lodging degree: 0 for all plants erect, 1 for <20% of the plants that became lodged, 2 for 20–40%, 3 for 40–60%, 4 for 60–80%, and 5 for 80–100% (few plants erect). The culm length was measured on 10 hills per plot. The weight of the straw and panicles were measured for 20 hills per plot. The experiment was performed on three plots.

**Table 4:** Cd concentration and amount of Cd accumulated in rice grown under early drainage water management in two farmers’ fields.

	Variety	Cd Concentration		Amount of Cd That Accumulated *		Total Amount of Cd that Accumulated in Aerial Parts **
		Straw	Panicle	Straw	Panicle	
		(mg kg^−1^)	(mg kg^−1^)	(mg m^−2^)	(mg m^−2^)	(mg m^−2^)
Field A	TJN25-11	24.5 ^a^ ± 2.9	37.4 ^a^ ± 5.5	24.2 ^a^ ± 1.7	12.3 ^a^ ± 0.7	36.5 ^a^ ± 2.2
	TJTT8	19.7 ^a^ ± 2.6	26.4 ^a^ ± 4.3	18.2 ^ab^ ± 2.4	6.1 ^b^ ± 0.3	24.3 ^b^ ± 2.3
	Cho-ko-koku	18.2 ^a^ ± 3.4	11.2 ^b^ ± 4.7	12.0 ^b^ ± 3.3	6.3 ^b^ ± 1.4	18.3 ^b^ ± 4.8
Field B	TJN25-11	21.1 ^a^ ± 1.4	27.9 ^a^ ± 2.4	16.3 ^a^ ± 1.7	4.2 ^a^ ± 0.2	20.5 ^a^ ± 1.6
	TJTT8	15.0 ^b^ ± 1.3	15.8 ^b^ ± 2.4	11.8 ^b^ ± 1.8	2.2 ^b^ ± 0.4	14.0 ^b^ ± 2.0
	Cho-ko-koku	12.8 ^b^ ± 0.7	5.7 ^c^ ± 0.4	7.9 ^c^ ± 0.8	1.9 ^b^ ± 0.4	9.7 ^c^ ± 1.1

The data are the means ± standard deviations (*n* = 3). The different letters show statistically significant differences between varieties at the 5% level of significance according to the Tukey test. The experiment included three repetitions. *: Amount of Cd that accumulated in each tissue, calculated by multiplying the Cd concentration by dry weight (cited from Table 3). **: Total amount of Cd that accumulated, calculated from sum of the amounts of Cd in the straw and panicles.

**Table 5:** Changes in soil Cd concentrations before and after planting and phytoextraction efficiency.

	Variety	Soil Cd Concentration (0.1 M HCl Extractable)		Average Amount of Cd in the Soil			Total Amount of Cd That Accumulated in Aerial Parts (F) ***	Phytoextraction Efficiency	
		Before Planting (A)	After Planting (B)	Before Planting (C) *	After Planting (D) **	Reduction (E = C − D)		Actual Reduction Rate of Soil Cd ^#^	Theoretical Reduction Rate of Soil Cd ^$^
		(mg kg^−1^)	(mg kg^−1^)	(mg m^−2^)	(mg m^−2^)	(mg m^−2^)	(mg m^−2^)	(%)	(%)
Field A	TJN25-11	0.33 ± 0.05	0.26 ± 0.02	49.5	39.0	10.5	36.5	21.2	73.7
	TJTT8	0.34 ± 0.06	0.30 ± 0.06	51.0	45.0	6.0	24.3	11.8	47.6
	Cho-ko-koku	0.32 ± 0.06	0.30 ± 0.05	48.0	45.0	3.0	18.3	6.3	38.1
Field B	TJN25-11	0.51 ± 0.00	0.40 ± 0.06	76.5	60.0	16.5	20.5	21.6	26.8
	TJTT8	0.53 ± 0.04	0.47 ± 0.08	79.5	70.5	9.0	14.0	11.3	17.6
	Cho-ko-koku	0.50 ± 0.06	0.46 ± 0.07	75.0	69.0	6.0	9.7	8.0	12.9

The soil Cd concentration data are the means ± standard deviations (*n* = 3). The other data are the means. *, **, (A or B) × (topsoil depth: 0–15 cm) × (soil bulk density: 1) × (area). ***, Each value is cited in Table 4. ^#^, (E/C) × 100. ^$^, (F/C) × 100.

## Data Availability

The data are contained within this article and the Supplementary Materials.

## Supplementary Materials

The following supporting information can be downloaded at https://www.mdpi.com/article/10.3390/plants11162178/s1. Figure S1: Ratio of straw Cd concentration in 31 TJN lines to that in Jarjan in field B. Figure S2: Physical map of SSR markers used for QTL analysis in F_2_ plants derived from Tachisugata and Nepal 555. Figure S3: Genotype of TJN25-11 at the nearest SSR marker to the putative QTLs. Table S1: Physicochemical properties of soils in fields A and B. Table S2: List of SSR markers used for MAS in the development of TJN lines.
