# Supplementary material for: QTL Pyramiding and Its Use in Breeding for Increasing the Phytoextraction Efficiency of Soil Cd via High-Cd-Accumulating Rice
Source: Plants (Basel). 2022 Aug 22;11(16):2178. doi: 10.3390/plants11162178 (PMC9415887; doi:10.3390/plants11162178)
Supplement: Supplementary file 1 [file plants-11-02178-s001.zip › plants-1848133-supplementary.pdf]

Supplementary data

QTL pyramiding and its use in breeding for increasing the phytoextraction efficiency of soil Cd via high-Cd-accumulating rice

Tadashi Abe[1], Masashi Ito[2], Ryuichi Takahashi[2], Toshimitsu Honma[3], Masato Kuramata[1], and Satoru Ishikawa[1]*

[1] Institute for Agro-Environmental Sciences, National Agriculture and Food Research Organization (NARO), Tsukuba, 305-8604, Japan.

[2] Akita Prefectural Agricultural Experiment Station, Akita, 010-1231, Japan.

[3] Niigata Agricultural Research Institute, Nagaoka, 940-0826, Japan.

* Correspondence: isatoru@affrc.go.jp

Figure S1. Ratio of straw Cd concentration in 31 TJN lines to that in Jarjan in field B.

Figure S2. Physical map of SSR markers used for QTL analysis in F_2 plants derived from Tachisugata and Nepal 555 and locations of *qHCd2* and *qHCd6* detected in this study.

Figure S3. Genotype of TJN25-11 at the nearest SSR marker to the putative QTLs (*qHCd2* and *qHCd6*).

Table S1. Physicochemical properties of soils in fields A and B.

Table S2. List of SSR markers used for MAS in the development of TJN lines and used for detecting *qHCd2* and *qHCd6* present in TJN25-11.

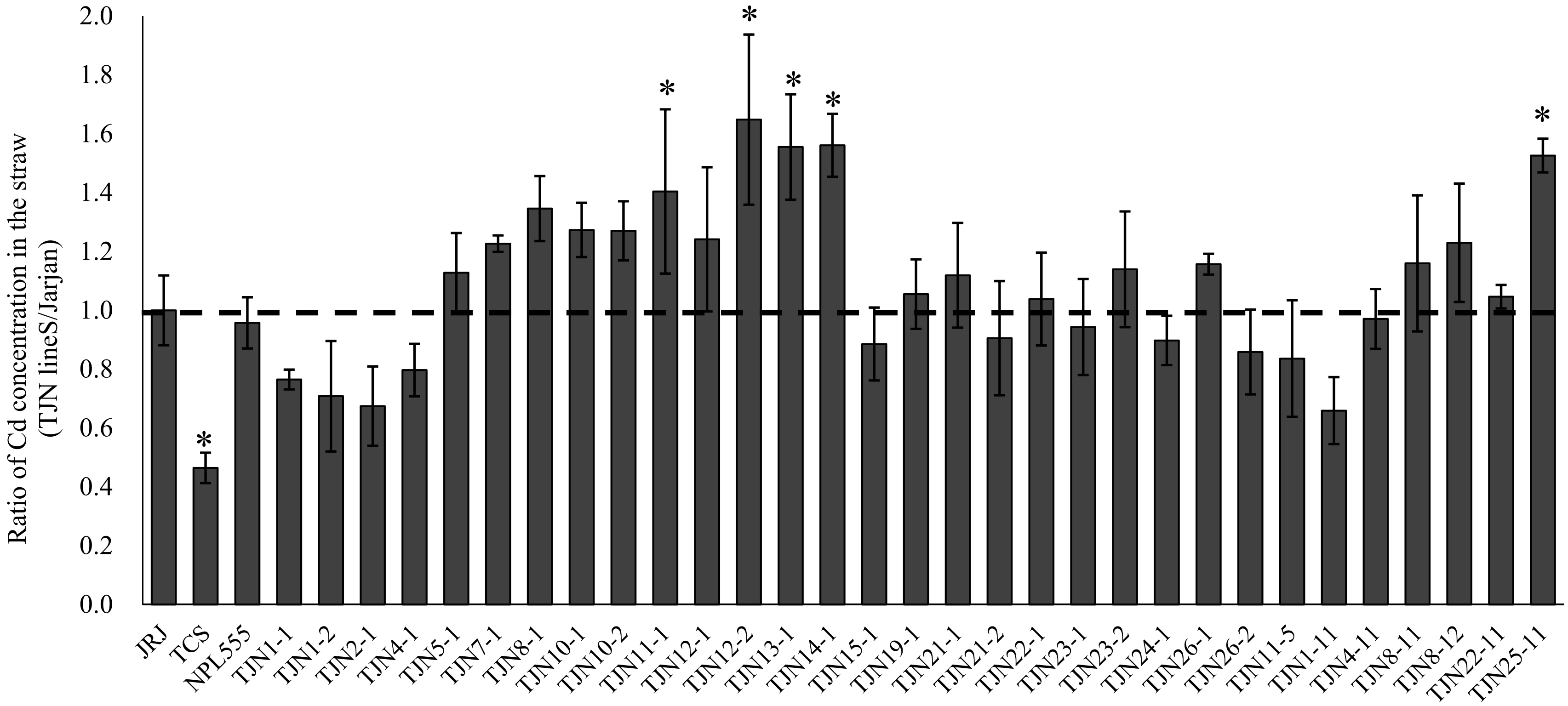

Figure S1. Ratio of straw Cd concentration in 31 TJN lines to that in Jarjan in field B.

The black asterisks show significant differences from Jarjan according to the Dunnett method ($p = 0.05$). The error bars indicate the standard deviations (n=3). JRJ: Jarjan, TCS: Tachisugata, NPL555: Nepal 555.

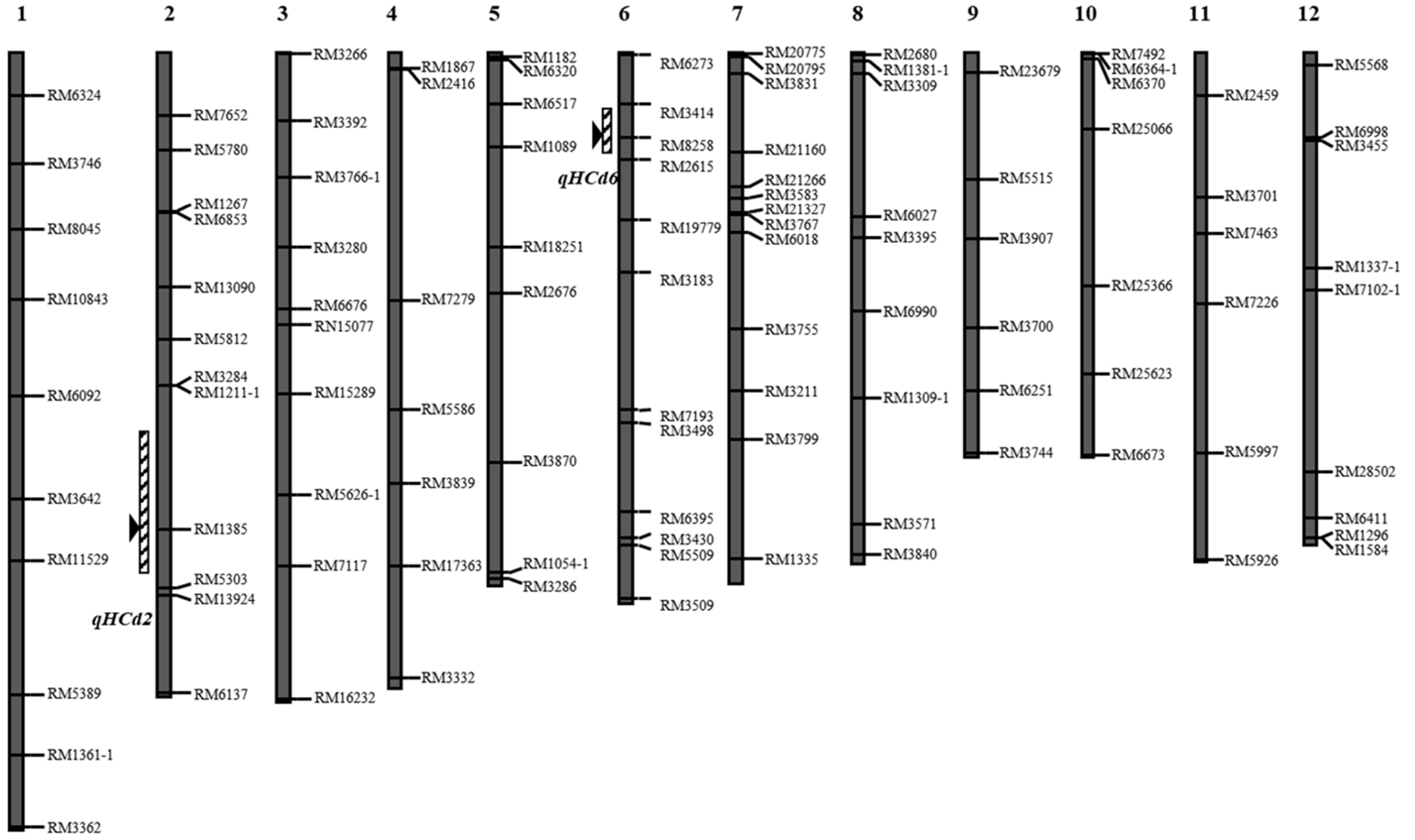

Figure S2. Physical map of SSR markers used for QTL analysis in F_2 plants derived from Tachisugata and Nepal 555 and locations of *qHCd2* and *qHCd6* detected in this study. The striped bars indicate the locations of *qHCd2* and *qHCd6*. The arrowheads indicate the nearest positions with the highest LOD values.

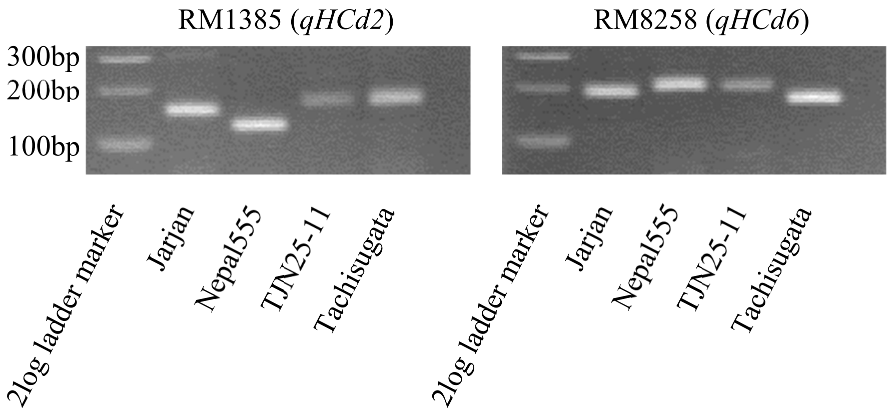

Figure S3. Genotype of TJN25-11 at the nearest SSR marker to the putative QTLs (*qHd2* and *qHCd6*). TJN25-11 carries the Tachisugata allele of *qHCd2* and the Nepal 555 allele of *qHCd6*.

Table S1. Physicochemical properties of soils in fields A and B.

	Soil classification	Texture	Sand content	pH	Total C	Total N	CEC	Exchangeable base			Available phosphate	Phosphate absorption coefficient	Cd 0.1 M HCl
								Ca	Mg	K			
			(%)	H_2O	(g kg^{-1})	(g kg^{-1})	($cmol_c$ kg^{-1})	($cmol_c$ kg^{-1})			(mg P_2O_5 kg^{-1})	(g P_2O_5 kg^{-1})	(mg kg^{-1})
Field A	Corse-textured mottled gley lowland soil	Sandy loam	80.0	6.55	14.2	1.2	8.6	6.2	0.9	0.3	414	4.4	0.33
Field B	Mottled gley lowland soil	Sandy loam	48.2	5.42	43.5	3.9	23.8	9.4	3.4	0.4	435	7.8	0.51

The soil type was based on the classification for cultivated soil in Japan (Classification Committee of Cultivated Soils 1996: Classification of Cultivated Soil in Japan – third Approximation. National Institute for Agro-Environmental Science, Tsukuba). The soil Cd concentrations were analyzed by using soils before planting in 2016. The other soil properties were analyzed by using soils before planting in 2014.

Table S2 List of SSR markers used for MAS in the development of TJN lines and used for detecting *qHCd2* and *qHCd6* present in TJN25-11.

Purpose	Locus name	Primer		Chr.	Traits	Target gene or QTL	Reference for gene or QTL
		Forward	Reverse				
MAS	RM5389	TCTTGCATGAGAGCCAACAC	GCTATTGCGCGAGATTATCC	1	shattering	*qSH1*	[26]
	RM1361-1	TGGTACAAGATGCAAGCGTC	GCCAACTTTCTCGTGCACAG				
	RM6728	GGGTATGTGTCGCTATTTTA	GAAATCTGGAATTTTCCCTA	7	brown pericarps	*Rc*	[25]
	RM1253	CTGAACTTGCCTGAGAACTC	GACGACCTCTCCATGCTCG				
	RM21320	CGTGCAACCCTATATGTAGA-TTGTGG	GGAGCCCGGAGTAATTTCTA-AAGC		high Cd	*HMA3*	[15] [21]
QTLs	RM1385	GACAGGTAAGGTGTGGTGGT-AAGG	AAACCTTTCTCAAACGCACA-CG	2	high Cd	*qHCd2*	this study
	RM8258	GTGCAGGACCCTCTATCTGT-GC	GTCGGTCTGACCACATGAAG-ACC	6	high Cd	*qHCd6*	this study

Reference for SSR markers: [27-28]
